# The association of blood lead levels and renal effects may be modified by genetic combinations of Metallothionein 1A 2A polymorphisms

**DOI:** 10.1038/s41598-020-66645-y

**Published:** 2020-06-15

**Authors:** Chen-Cheng Yang, Chia-I Lin, Su-Shin Lee, Chao-Ling Wang, Chia-Yen Dai, Hung-Yi Chuang

**Affiliations:** 1 Graduate Institute of Medicine, College of Medicine, Kaohsiung Medical University, Kaohsiung, Taiwan; 2 Department of Occupational and Environmental Medicine, Kaohsiung Municipal Siaogang Hospital, Kaohsiung, Taiwan; 3 Department of Family Medicine, Kaohsiung Municipal Siaogang Hospital, Kaohsiung, Taiwan; 4 Department of Occupational and Environmental Medicine, Kaohsiung Medical University Hospital, Kaohsiung, Taiwan; 5 Health Management Center, Kaohsiung Municipal Ta-Tung Hospital, Kaohsiung, Taiwan; 6 Center for Stem Cell Research, Kaohsiung Medical University, Kaohsiung, Taiwan; 7 Department of Public Health, College of Health Sciences, Kaohsiung Medical University, Kaohsiung, Taiwan

**Keywords:** Occupational health, Health occupations, Genetics research

## Abstract

Metallothionein (MT) is a protein with function of heavy metal detoxification. However, studies about how single nucleotide polymorphisms (SNPs) of MT genes influence lead nephropathy are relatively scarce. Therefore, our aim is to investigate the association between blood lead levels and renal biomarkers and to study whether this association is influenced by the combination of MT1A and MT2A SNPs. Blood lead, urinary uric acid (UA), and urinary N-acetyl-beta-d-glucosaminidase (NAG) levels were analyzed from 485 participants. Genotyping were performed on MT1A SNPs (rs11640851 and rs8052394) and MT2A SNPs (rs10636 and rs28366003). The combined MT1A 2A SNPs were divided into 16 groups. Among renal biomarkers, urinary UA was negatively significant associated with the time-weighted index of cumulative blood lead (TWICL), while urinary NAG was positively significant with TWICL. Furthermore, the association between urinary UA and TWICL was significantly modified by group 6 of combined SNPs (MT1A 2 A SNPs combination were AAAGGGAA, ACAGGGAA, and ACGGGGAA). In conclusion, the negative association of urinary UA and TWICL is modified by group 6, which means participants of group 6 are more susceptible to lead nephrotoxicity.

## Introduction

Lead is an indispensable material in industry but represents an important issue in public health. It causes a variety of harmful effects on multiple organs, from hematopoietic dysfunction, adverse neuro-behavioral health effects, respiratory dysfunction, liver toxicity, hypertension and cardiovascular diseases to impaired renal function^1–10^. Since the late 1920s, a relationship between childhood lead exposure and lead-induced nephropathy has been found in Queensland, Australia^11^. Moreover, accelerated deterioration of chronic renal insufficiency is influenced even at low levels of lead exposure^12^.

Metallothioneins (MTs) are high cysteine-containing, low molecular weight proteins that were initially found in the equine renal cortex in 1957^13^. The functions of these proteins include the maintenance of metal equilibria that protect against heavy metal ion toxicity and oxidative damage^14–18^. Tokar *et al*. found that inorganic lead exposure in early life induces testicular teratoma and renal and urinary bladder preneoplasia in MT-null mice^19^. The MT gene family in mammals consists of four subfamilies, from MT1 to MT4^20,21^. Human MT genes are located on chromosome 16q13^22^. Bylander *et al*. found that exposure of human proximal tubule cells to CdCl_2_ induces additional MT1A mRNA expression^23^. Lei *et al*. noted that MT1A polymorphisms were associated with high exposure levels of cadmium^24^. Yang *et al*. showed that MT1A SNPs may influence urinary UA and NAG excretion in chronic lead exposure^25^. Hattori *et al*. found that MT2A rs28366003 is a risk factor of chronic kidney disease and diabetes mellitus in a community-based cohort population^26^. Tekin *et al*. suggested that in pregnant participants, different MT2A polymorphisms might be associated with blood lead levels^27^. Kayaalti *et al*. revealed that MT2A rs28366003 GG genotype carriers might be more influenced by cadmium, lead and zinc toxicity^28^. Shokrzadeh *et al*. found the MT-2A gene polymorphisms (rs1610216, rs28366003) were associated with the risk of adenocarcinoma^29^. Chen *et al*. found that the MT4 rs396230 G variants were more susceptible to lead toxicity on kidney^30^.

However, there are scarce investigations about the influence of MT1A and 2 A polymorphisms on renal function in chronic occupational lead exposure. Our aim is to investigate the association of blood lead levels and renal biomarkers in chronic occupational lead exposure and to study whether the association was affected by combinations of MT1A and MT2A polymorphisms.

## Results

Combination of the 4 SNPs in MT1A and MT2A, there should be 81 genotypes (3^4^). However, carriers of certain types, such as MT1A rs11640851 CC- MT1A rs8052394 AA- MT2A rs10636 GG- MT2A rs28366003 GG (CCAAGGGG), did not exist. Thus, in our study, there were only 44 genotypes existed. We classified our participants based on wild-type and variant-type carriers into 16 groups (Table 1). For example, MT1A rs11640851, we regarded the CC allele as wild type due to the largest numbers, while CA and AA carriers were regarded as variant types. The same principle of classification was also applied in MT1A rs8052394, MT2A rs10636 and rs28366003, which were regarded rs8052394 AA, rs10636 GG and rs28366003 AA as wild types, respectively, all wild types combination belonged to group 1, the pure wild-type genetic carriers (CCAAGGAA), 20 participants as reference. For only one variant, such as group 2 in Table 1, AAAAGGAA, ACAAGGAA, MT1A rs11640851 was variant and the other 3 SNPs were wild types. The other groups were according to above principle and shown in Table 1.

**Table 1:** Sixteen groups of MT1A and MT2A SNP combinations of MT1A rs11640851, MT1A rs8052394, MT2A rs10636, and MT2A rs28366003, according to wild types and variant types.

Group	Principle of the combination	MT1A2A combination	Case number	Percentage (%)
1	Wild-Wild-Wild-Wild	CCAAGGAA	20	4.1
2	Variant-Wild-Wild-Wild	AAAAGGAA, ACAAGGAA	77	15.9
3	Wild-Variant-Wild-Wild	CCAGGGAA, CCGGGGAA	46	9.5
4	Wild-Wild-Variant-Wild	CCAACCAA, CCAAGCAA	14	2.9
5	Wild-Wild-Wild-Variant	CCAAGGAG	32	6.6
6	Variant-Variant-Wild-Wild	AAAGGGAA, ACAGGGAA, ACGGGGAA	55	11.3
7	Variant-Wild-Variant-Wild	AAAACCAA, AAAAGCAA, ACAACCAA, ACAAGCAA	67	13.8
8	Variant-Wild-Wild-Variant	AAAAGGAG, ACAAGGAG	6	1.2
9	Wild-Variant-Variant-Wild	CCAGGCAA, CCGGGCAA	23	4.7
10	Wild-Variant-Wild-Variant	CCAGGGAG, CCAGGGGG	5	1.0
11	Wild-Wild-Variant-Variant	CCAACCAG, CCAACCGG, CCAAGCAG	5	1.0
12	Variant-Variant-Variant-Wild	AAAGCCAA, AAAGGCAA, AAGGGCAA, ACAGCCAA, ACAGGCAA, ACGGGCAA	79	16.3
13	Variant-Variant-Wild-Variant	ACAGGGAG	6	1.2
14	Variant-Wild-Variant-Variant	AAAACCAG, AAAAGCAG, ACAACCAG, ACAAGCAG	28	5.8
15	Wild-Variant-Variant-Variant	CCAGCCAG, CCAGCCGG, CCAGGCAG, CCGGCCAG, CCGGGCAG	10	2.1
16	Variant-Variant-Variant-Variant	AAGGGCAG, ACAGCCAG, ACAGGCAG, ACGGGCAG	12	2.5
Total			485	100

Table 2 shows the descriptive analysis of the demographic characteristics, lead parameters, renal biomarkers, and SNP distributions. First, the mean participant age was 42.40 years, with a standard deviation (SD) of 7.99 years, while the mean job duration was 12.94 years, with standard deviation of 7.80 years. Second, the mean and SD of body weight were 62.09 ± 11.18 kg, respectively, and the mean and SD of BMI were 23.49 ± 3.31 kg/m^2^, respectively. The mean and SD of systolic blood pressure were 123.19 ± 17.01 mmHg, respectively, and were 76.81 ± 10.96 mmHg, respectively, for diastolic blood pressure. Third, the mean and SD of the current blood lead level, index of cumulative lead (ICL), and time-weighted ICL (TWICL) were 22.30 ± 13.34 μg/dL, 339.82 ± 304.62 μg × yr/dL, and 24.98 ± 12.71 μg/dL, respectively. Fourth, the renal parameters, the mean and SD of serum UA, serum creatinine, urinary creatinine, urinary UA and urinary NAG were 6.37 ± 1.53 mg/dL, 0.95 ± 0.22 mg/dL, 190.11 ± 97.62 mg/dL, 36.88 ± 16.63 mg/gCr, and 3.13 ± 1.88 mg/gCr, respectively. The participants included 212 females (43.71%) and 273 males (56.29%). Moreover, 37.73% (183 subjects) of all participants were smokers and 15.46% (75 subjects) consumed alcohol. Finally, all MT1A and 2 A genotype frequencies were consistent with the Hardy-Weinberg equilibrium. (detailed data, please refer to Supplementary Table S1)

**Table 2:** Descriptive analysis of the demographic characteristics, biomarker levels, and MT1A and MT2A SNPs.

	Mean ± SD	Medium	IQR (25%-75%)
Age (years)	42.40 ± 7.99	43.20	37.04–48.55
Job duration (years)	12.94 ± 7.80	11.23	7.03–16.17
Body height (cm)	162.28 ± 8.21	162.50	156.00–168.70
Body weight (kg)	62.09 ± 11.18	61.20	53.45–70.20
Body mass index (kg/m^2^)	23.49 ± 3.31	23.32	21.10–25.40
Systolic blood pressure (mmHg)	123.19 ± 17.01	121.00	112.00–133.00
Diastolic blood pressure (mmHg)	76.81 ± 10.96	76.00	69.00–83.00
Current blood lead (μg/dL)	22.30 ± 13.34	21.10	11.80–31.00
Index of cumulative lead (μg/ × yr/dL)	339.82 ± 304.62	259.90	116.70–449.34
Time-weighted ICL (μg/dL)	24.98 ± 12.71	24.59	15.32–33.03
Serum uric acid (mg/dL)	6.37 ± 1.53	6.20	5.20–7.30
Serum creatinine (mg/dL)	0.95 ± 0.22	0.90	0.80–1.10
Urinary creatinine (mg/dL)	190.11 ± 97.62	174.00	127.00–241.00
Urinary uric acid (mg/g Cr)	36.88 ± 16.63	34.87	24.87–47.18
Urinary NAG (mg/g Cr)	3.13 ± 1.88	2.66	1.94–3.78
Gender	Case Number (%)		
Female (%)	212 (43.71%)		
Male (%)	273 (56.29%)		
Smoking	Case Number (%)		
Yes (%)	183 (37.73%)		
No (%)	302 (62.27%)		
Drinking	Case Number (%)		
Yes (%)	75 (15.46%)		
No (%)	410 (84.53%)		
MT1A, rs11640851	Case Number (%)	(HWE, p = 0.447)	
AA	99 (20.41%)		
AC	231 (47.63%)		
CC	155 (31.96%)		
MT1A, rs8052394	Case Number (%)	(HWE, p = 0.631)	
AA	249 (51.34%)		
AG	194 (40.00%)		
GG	42 (8.66%)		
MT2A, rs10636	Case Number (%)	(HWE, p = 0.508)	
GG	247 (50.93%)		
GC	194 (40.00%)		
CC	44 (9.07%)		
MT2A, rs28366003	Case Number (%)	(HWE, p = 0.179)	
AA	381 (78.56%)		
AG	101 (20.82%)		
GG	3 (0.62%)		

IQR: Interquartile range; HWE: Hardy-Weinberg equilibrium.

In Table 3, in addition to TWICL and other potential confounders, we arranged the 16 groups of MT1A2A SNP combinations for the regression analysis of renal parameters. We assumed group 1, the pure wild-type combination, as the reference group. For the 16 groups of MT1A2A combinations, we arranged multi-comparisons with Bonferroni corrections with α error (α error = 0.05)^31,32^. Bonferroni-corrected adjusted p-values were defined as α divided by the number of SNPs, which meant the significant difference in SNP combination groups was adjusted, with p-values less than 0.003125 (0.05/16). For serum creatinine, males and females were significantly different, in which the serum creatinine was 0.32 mg/dL higher in males than in females, p-value <0.0001. There was no significant difference in group 2 to group 16 in comparison to group 1. Serum uric acid (UA) was positively associated with males and with higher BMI, with increases of 1.47 mg/dL and 0.099 mg/dL, respectively. Urinary UA showed significantly lower levels in males than in females, with a difference of −4.77 mg/g Cr. Meanwhile, groups 6 was significantly (p = 0.0008) and group 12 was slightly (p = 0.0047) negatively associated with urinary UA levels, which decreased by 13.52 mg/g Cr and 10.97 mg/g Cr, respectively. However, group 5 was slightly positively (p = 0.0047) associated with urinary UA levels (increasing 12.47 mg/g Cr). TWICL had a significant negative association with urinary UA without modification of genotype (Data shown in Supplementary Table S2). However, taking 16 groups as covariates in the regression model, the negative association between TWICL and urinary UA became non-significantly. On the other hand, there was a significant increase in urinary NAG level of 0.016 mg/g Cr, 0.047 mg/g Cr and 0.50 mg/g Cr for TWICL, age and smoking, respectively. Moreover, males had a lower urinary NAG level than females, which was 0.74 mg/g Cr; however, no genotype modification effects were found.

**Table 3:** Regression model of renal biomarkers predicted by the TWICL, 16 types of MT 1A and 2A SNP combination, and other potential confounders.

	Serum creatinine (mg/dL)		Serum uric acid (mg/dL)		Urinary uric acid (mg/g Cr)		Urinary NAG (mg/g Cr)	
	ß (SE)	p-value	ß (SE)	p-value	ß (SE)	p-value	ß (SE)	p-value
Time-weighted ICL (μg/dL)	−0.0006 (0.0007)	0.38	0.0081 (0.0053)	0.13	−0.054 (0.065)	0.41	0.016 (0.0076)	0.043
Group 1	—	—	—	—	—	—	—	—
Group 2	0.020 (0.042)	0.63	0.48 (0.31)	0.13	−0.019 (3.83)	1.00	0.010 (0.45)	0.98
Group 3	−0.0082 (0.045)	0.86	0.40 (0.34)	0.25	0.040 (4.14)	0.99	0.30 (0.49)	0.54
Group 4	0.079 (0.058)	0.17	0.78 (0.44)	0.076	−1.90 (5.31)	0.72	0.43 (0.62)	0.49
Group 5	−0.0032 (0.048)	0.95	0.29 (0.36)	0.42	12.47 (4.39)	0.0047	−0.17 (0.52)	0.75
Group 6	0.012 (0.044)	0.78	0.48 (0.33)	0.15	−13.52 (4.00)	0.0008^*^	0.030 (0.47)	0.95
Group 7	0.030 (0.043)	0.49	0.33 (0.32)	0.31	−9.20 (3.91)	0.019	0.033 (0.46)	0.94
Group 8	0.044 (0.078)	0.57	−0.12 (0.59)	0.84	8.92 (7.16)	0.21	−0.33 (0.84)	0.70
Group 9	0.0038 (0.052)	0.94	0.14 (0.39)	0.71	−9.70 (4.74)	0.041	0.45 (0.56)	0.42
Group 10	−0.076 (0.084)	0.37	−0.47 (0.63)	0.46	3.94 (7.68)	0.61	−0.098 (0.90)	0.91
Group 11	−0.017 (0.084)	0.84	0.65 (0.63)	0.30	1.92 (7.64)	0.80	−0.19 (0.90)	0.83
Group 12	−0.0023 (0.042)	0.96	0.53 (0.32)	0.10	−10.97 (3.87)	0.0047	0.90 (0.45)	0.047
Group 13	0.066 (0.078)	0.40	1.20 (0.59)	0.041	0.76 (7.12)	0.92	−0.44 (0.84)	0.60
Group 14	0.020 (0.049)	0.69	0.23 (0.37)	0.54	−1.55 (4.46)	0.73	0.31 (0.52)	0.56
Group 15	−0.061 (0.064)	0.34	0.21 (0.48)	0.66	4.32 (5.89)	0.46	0.54 (0.69)	0.43
Group 16	0.030 (0.061)	0.62	1.034 (0.46)	0.025	−8.33 (5.60)	0.14	0.59(0.66)	0.37
Gender(male)	0.32 (0.021)	<0.0001	1.47 (0.16)	<0.0001	−4.77 (1.94)	0.014	−0.74 (0.23)	0.0013
Age (year)	−0.0002 (0.0010)	0.88	−0.012 (0.0077)	0.11	0.033 (0.094)	0.73	0.047 (0.011)	<0.0001
BMI (kg/m^2^)	0.0011 (0.0024)	0.65	0.099 (0.018)	<0.0001	0.46 (0.22)	0.036	0.035 (0.026)	0.17
Smoke	−0.032 (0.021)	0.13	−0.034 (0.16)	0.83	1.87 (1.96)	0.34	0.50 (0.23)	0.031
Drink	0.0070 (0.024)	0.77	0.014 (0.18)	0.94	1.17 (2.16)	0.59	0.19 (0.25)	0.45
Constant	0.77 (0.072)	<0.0001	3.15 (0.54)	<0.0001	31.99 (6.58)	<0.0001	−0.13 (0.77)	0.8660

^*^adjusted p−value < 0.003125: adjusted by Bonferroni correction (α/number of SNPs).

**Figure 1:**
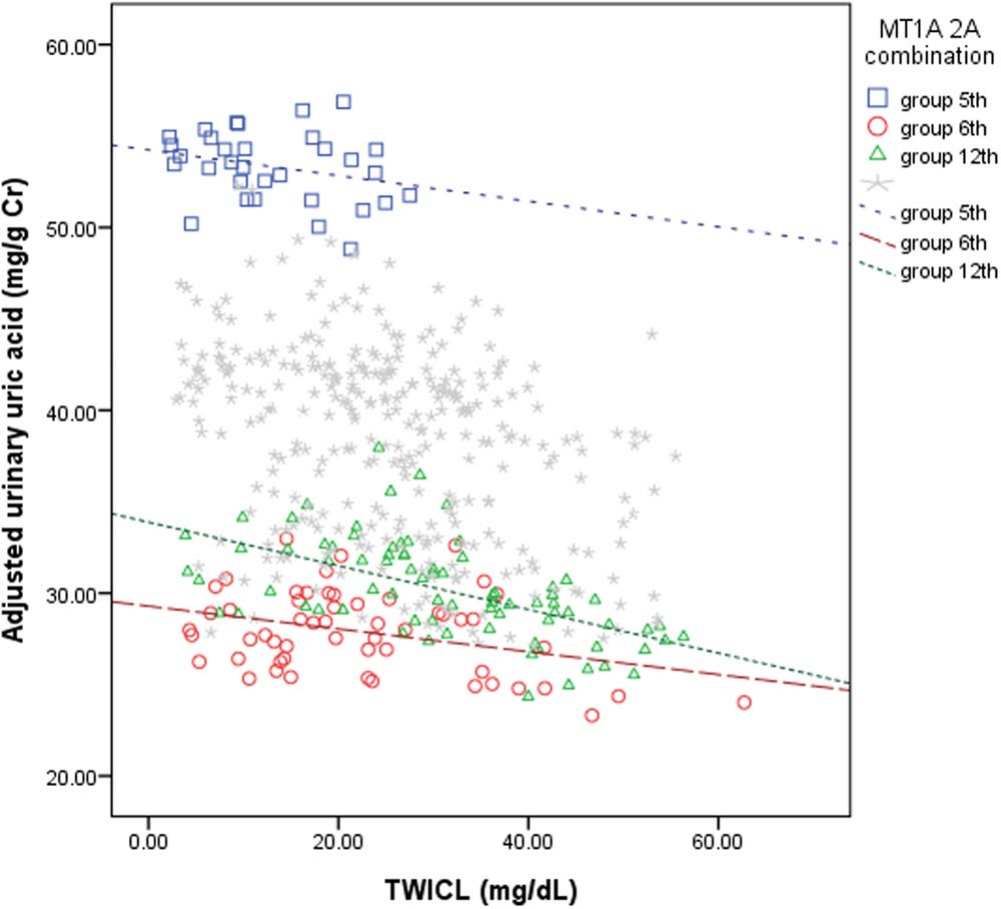
The association of urinary UA and TWICL between group 5, group 6, and group 12 of MT1A2A genetic combinations. Lead-influenced urinary UA levels by the association of increasing blood lead levels with decreasing urinary UA levels. Furthermore, the urinary UA was significantly decreased in group 6 (β -13.52, p-value 0.0008) and was weakly decreased in group 12 (β -10.97, p-value 0.0047), while it was slightly increased in group 5 (β 12.47, p-value 0.0047).

**Figure 2:**
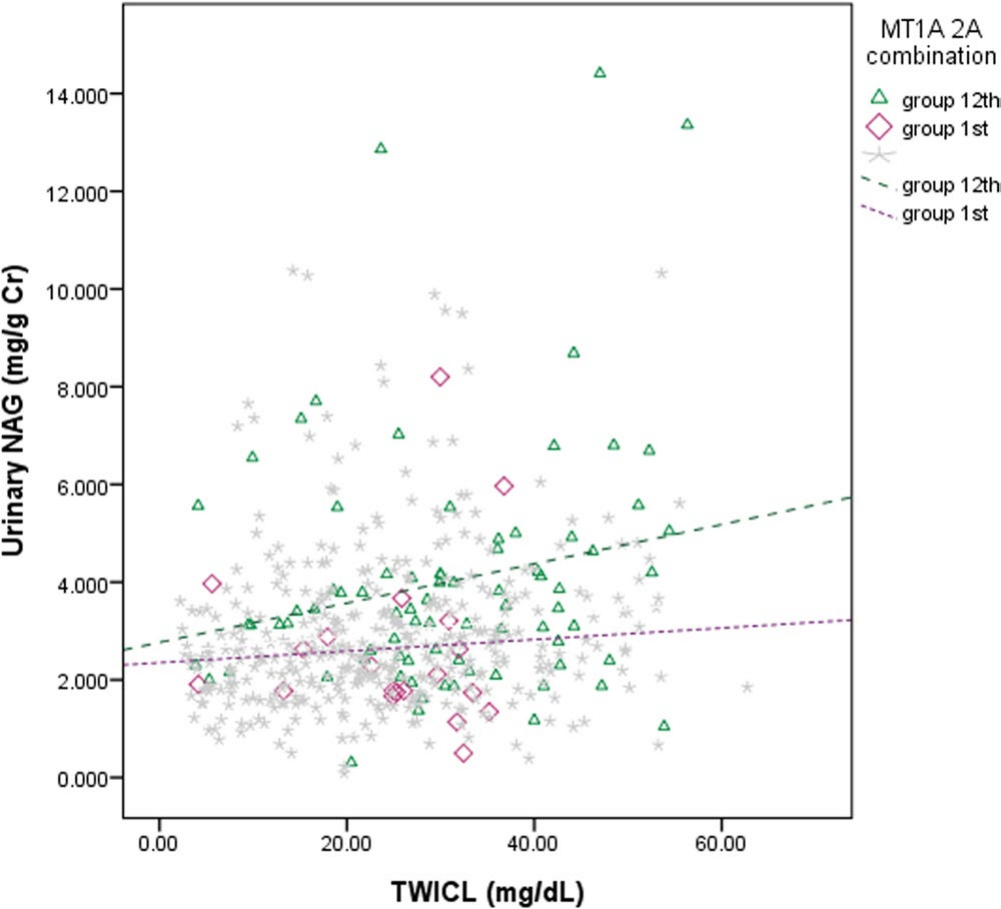
The association of urinary NAG and TWICL between group 12 and the reference group. Lead influenced urinary NAG, which is shown in Fig. 2, by the association of increasing blood lead levels with increasing urinary NAG, especially in group 12 (β 0.90, p-value 0.047), which conveys a slightly higher susceptibility to elevated lead concentration than the reference group.

Figure 1 shows the association of urinary UA and TWICL between groups 5, 6, and 12 in the group with MT1A2A genetic combinations. Lead was associated with urinary UA levels, which revealed the association between increasing blood lead levels and lower urinary UA without any genotypes adjusted (Data shown in Supplementary Table S2). However, when adjusted for genotype, urinary UA was significantly decreased in groups 6 (β −13.52, p-value 0.0008) and was weakly decreased in group 12 (β −10.97, p-value 0.0047). Meanwhile, urinary UA was weakly higher in group 5 (β 12.47, p-value 0.0047). On the other hand, Fig. 2 shows the association of urinary NAG and TWICL between group 12 and the reference group. Lead influenced urinary NAG by the association of slightly increased blood lead level with higher urinary NAG, especially in group 12 (β 0.90, p-value 0.047).

## Discussion

Lead containing gasoline has been prohibited since 2000 in Taiwan, so battery factory exposure has become one of the main lead-exposure sources. This study found two urinary biomarkers that may be alternative indicators of renal lead toxicity, urinary NAG and urinary UA are more easily measured by the non-invasive procedure of urine collection. Urinary NAG revealed a positive association with increased TWICL, regardless of whether the adjustment occurred before or after the 16 groups of SNP combinations were created. Our finding corresponds to several studies about urinary NAG and lead. Ehrlich *et al*. conducted a urinary marker analysis in 199 participants among South African battery factory workers, and their investigation showed that urinary NAG was positively associated with current and historical blood lead levels^33^. Not only in adults but also in adolescents, Sonmez *et al*. tested 39 auto repairmen, 13 battery factory employees and 29 health rural subjects for blood lead levels and urinary NAG levels; the results showed significantly higher blood lead levels and higher urinary NAG levels in battery workers than in auto repairmen. Similar results were also found between auto repairmen and non-lead-exposed healthy rural adolescents^34^. However, Sonmez did not discuss whether there were dose-response relationships between lead and urinary NAG in their participants. Oktem *et al*. tested 79 lead-exposed adolescent auto repair workers in Turkey and 71 healthy control subjects for urinary NAG and lead toxicity. The urinary NAG levels of auto repairmen were significantly higher than those of the reference group and were positively associated with blood lead levels^35^. Hence, urinary NAG should be considered an indicator of renal toxicity in lead exposure.

Furthermore, UA is another biomarker of lead toxicity because lead may cause inhibited UA secretion. In a study of 111 healthy subjects, blood lead level was positively associated with serum UA level^36^. Ahmed *et al*. carried out a cross-sectional study on lead-exposed workers in a battery factory. Compared with non-exposed groups, serum UA was significantly higher in lead-exposed participants^37^. Although no significant finding between serum UA and lead in our regression model, we found that urinary UA has a significant negative association as TWICL increased before MT1A2A SNP combination adjustment, and the association disappeared after adjustment, which suggest combinations of MT1A2A SNPs play an important role in urinary UA secretion with lead-induced renal toxicity.

MTs are cysteine-rich, low molecular weight proteins with heavy metal detoxification and antioxidant effects^38^. Compared with the wild-type mice, MT-null mice showed dose-dependent nephromegaly as the lead treatment dose increased. Furthermore, as the lead toxicity increased, MT-knockout mice did not form lead-induced inclusion bodies and had significantly increased lead accumulation in the kidneys^39^. Waalkes *et al*. conducted lead exposure in MT-null and wild-type mice, and the incidence of total renal proliferative lesions showed a clear dose-dependent relationship in both wide-type and MT-null mice. In the maximum dose-exposure group (4000 ppm), the incidence of renal cystic hyperplasia and carcinogenesis was significantly higher in MT-knockout mice than in wild-type mice^40^. These studies implied how the MT influence renal effects; however, how genetic polymorphisms affect lead toxicity has not been clarified.

However, no previous studies discussed how the MT1A and MT2A SNP combinations influenced lead nephrotoxicity. To our knowledge, this study is the first investigation about the relationship between lead and renal biomarkers with the influence of MT1A2A genetic combinations. Although the weak positive association between serum UA and TWICL, and the negative association between urinary UA and TWICL disappeared after adjusting for the 16 groups of MT1A2A SNP combination, the positive association between urinary NAG and TWICL was maintained. After adjusting for MT1A2A SNP combinations, urinary UA was decreased significantly in groups 6 and slightly in group 12, while urinary UA was increased weakly in group 5. This implies that the 16 groups of MT1A2A SNP combinations might modify the relationship between renal biomarkers and lead toxicity. It meant that groups 6 and 12 were more susceptible to lead toxicity in comparison to group 1, while group 5 might be less susceptible to lead toxicity than group 1. Figure 1 also revealed different genetic combinations have different slopes. It implied different genetic combinations modify the association between urinary UA and TWICL. The TWICL of group 12 was not significantly different from TWICL of other groups but TWICL of group 5. Based on the one-way analysis of variance (ANOVA) with Scheffe test (analysis not shown), we could not conclude that TWICL of group 12 (mean TWICL: 30.71 ug/dL) was strange. Thus, the polymorphisms did not likely affect blood lead, especially TWICL; furthermore, we had not measured the activity or levels of MT proteins among these SNP types, which further investigations are needed.

One of the limitations of our investigation is we did not measure the activities and levels of MT according to MT1A2A SNP combinations owing to the unavailability of extra biological samples. Moreover, many factors influence MT activities and levels, including age, gender, intake components, and other undiscovered factors. Second, we did not check environmental lead sources, such as air or soil lead levels; however, we regard the blood lead levels as an internal dose indicator of the exact absorption of lead from the environment to the human body.

## Conclusions

In conclusion, urinary uric acid is negatively significant associated with the TWICL, while urinary NAG is positively significant with TWICL. Urinary UA and urinary NAG levels should be considered as suitable indicators of lead nephrotoxicity. Furthermore, the negative association of urinary UA and TWICL is modified by group 6 (MT1A 2A SNPs combination are AAAGGGAA, ACAGGGAA, and ACGGGGAA), which means group 6 is the more susceptible to lead nephrotoxicity.

## Materials and Methods

### Participants and health examinations

**Figure 3:**
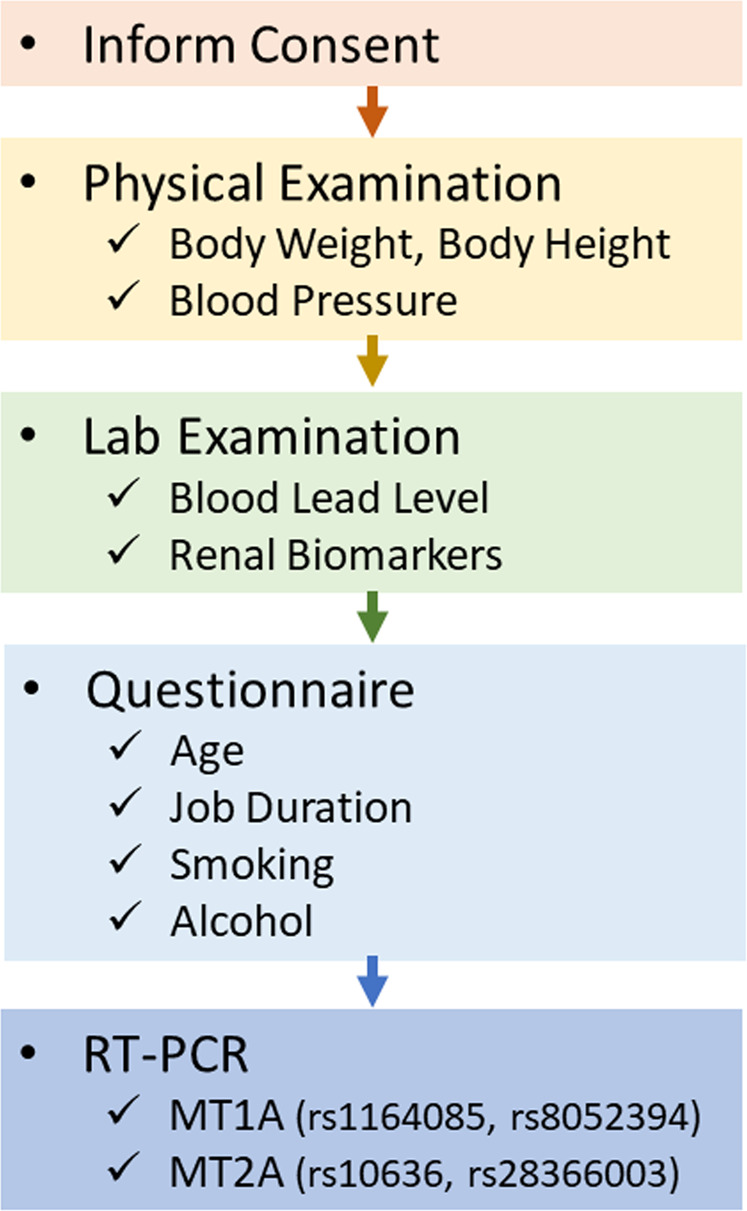
Schematic diagram of protocol.

Based on Taiwan Occupational Safety and Health Act^41^, lead-exposed workers should receive annual health examinations that include a physical examination, blood lead level test, and blood and urine routine examination. Furthermore, a short questionnaire was performed, including information regarding alcohol consumption and cigarette use. Drinking more than six alcoholic drink equivalent a week is defined as drinking, while currently smoking is defined as a smoker. Schematic diagram of Protocol was illustrated as Fig. 3.

This investigation was conducted during the annual health examination of lead-exposed workers from a battery factory after the participants’ informed consents were obtained (the institutional review board in Kaohsiung Medical University Hospital approval No: KMUHIRB-20120077); finally a total of 485 lead-exposed participants were recruited. All research was performed in accordance with relevant guidelines/regulations, and informed consent was obtained from all participants. Our samples were measured in the central laboratory of Kaohsiung Medical University Hospital.

### Blood lead level analysis and parameter calculation

Venous blood samples were collected by means of standard procedures. Standard procedure of blood lead analysis was shown in Supplementary Method about standard procedures of blood lead analysis online. Furthermore, we calculated the index of cumulative blood lead (ICL) and the time-weighted index of cumulative blood lead (TWICL). The calculation methods of the cumulative indexes were published elsewhere in a previous study^42^. The ICL was calculated by integrating blood lead levels over each participant’s employment time by the trapezoidal rule, which formula was calculated using Eq. (1).

1 $${\rm{ICL}}=\int {{\rm{PbB}}}_{{\rm{t}}}\Delta {\rm{t}}=\sum 0.5({{\rm{PbB}}}_{{\rm{i}}}+{{\rm{PbB}}}_{{\rm{i}}+1}){\Delta {\rm{t}}}_{{\rm{i}}}.$$

Where PbB_i_ and PbB_i+1_ represent the initial year and next year measurements of blood lead level, taken Δt years apart.

TWICL was calculated by dividing ICL by the summation of job time, which formula was calculated using Eq. (2).

2 $${\rm{TWICL}}=\{\sum 0.5({{\rm{PbB}}}_{{\rm{i}}}+{{\rm{PbB}}}_{{\rm{i}}+1}){\Delta {\rm{t}}}_{{\rm{i}}}\}/\sum {\Delta {\rm{t}}}_{{\rm{i}}}.$$

### Renal biomarker analyses and measurement of urinary UA and urinary NAG

Serum creatinine, serum UA and urinary UA were measured with a biochemistry automatic analyzer (Toshiba TBA c8000 Chemistry Analyzer, Toshiba Medical Systems Co., Tokyo) in the central laboratory of Kaohsiung Medicine University Hospital. All the laboratory technicians were blinded to the exposure status of the investigation subjects.

Urine concentrations of NAG were measured with the NAG assay kit from Diazyme (Diazyme Laboratories, Poway, CA). Briefly, we used the Diazyme NAG assay with a specific substrate, 2-methoxy-4-(2′-nitrovinyl)-phenyl 2-acetamido-2-deoxy–D-glucopyranoside (MNP-GlcNAc), which was hydrolyzed by NAG to become 2-methoxy-4-(2-nitrovinyl)-phenol product. Then, an alkaline buffer (sodium carbonate buffer; pH 10) was added into the sample, which was detected at 505 nm. Using this assay, no detectable reaction with other glycosidases were noted, and we had excellent sensitivity, with a linear range from 0 up to 200 U/l, with CV (coefficient of variation) <5%.

All the urine biomarkers were corrected for urinary creatinine levels, including the urinary UA and urinary NAG.

### Metallothionein genetic polymorphism analyses

We used the dbSNP database of the National Center for Biotechnology Information (http://www.ncbi.nlm.nih.gov/SNP/) and the HapMap database to search for genetic polymorphisms. It was found that the genotypes rs11640851 (A/C transition of the human MT1A gene, leading to an Asp27Thr amino acid substitution), rs8052394 (A/G transition of the human MT1A gene, leading to an Lys51 Arg amino acid change), MT2A rs10636 (G/C), located in the 3′ untranslated region, and MT2A rs28366003 (A/G), located in the 5′ untranslated region, have minor allele frequencies higher than 10% in the Han Chinese population; thus, these genes were selected for the genetic polymorphic analysis. Genotyping procedure was shown in Supplementary Method of MT1A and MT2A genotyping procedure online. The allele frequencies were determined by using ABI SDS software. The genotyping procedure was repeated on a random 10% of samples to confirm the results of the original run by the laboratory technicians, who were blinded to the original results. The estimated genotyping error rate was less than 1%.

### Statistical analyses

For the continuous variables, descriptive statistics were used to calculate the means, including blood lead levels, age, blood pressure, serum creatinine, serum UA, urinary UA and urinary NAG, and they were also used to calculate the dispersion, medium and interquartile ranges of these data. Categorical variables, such as gender, smoking and alcohol consumption, were treated with proportions. Nominal variables among groups were calculated with the Chi-squared test. Depending on the MT1A and MT2A genetic polymorphisms, the participants were divided into three categories. One-way ANOVA with post-hoc analyses of Scheffe’s test was performed to examine differences in blood lead levels, renal function parameters, and other confounders among the three categories^43^. A multiple linear regression analysis was used to evaluate the association of renal biomarkers with blood lead levels, while adjusting for the 16 groups of MT1A2A genetic polymorphism combinations and other potential confounders, such as age, gender, BMI, smoking and drinking status. We analyzed these data by IBM-SPSS version 20 and SAS version 9.4 statistical software, with α set at 0.05 and two-tailed; thus, p-values less than 0.05 were considered significant.

## Supplementary information

Supplementary Information.

## Data Availability

The authors declare that all data supporting the findings of this study are available within the article or from the corresponding author upon reasonable request.
