# Supplementary material for: The association of blood lead levels and renal effects may be modified by genetic combinations of Metallothionein 1A 2A polymorphisms
Source: Sci Rep. 2020 Jun 15;10:9603. doi: 10.1038/s41598-020-66645-y (PMC7295782; doi:10.1038/s41598-020-66645-y)
Supplement: Supplementary file 1 — Supplementary Information. [file 41598_2020_66645_MOESM1_ESM.docx]

**The association of blood lead levels and renal effects may be modified by genetic combinations of Metallothionein 1A 2A polymorphisms**

Chen-Cheng Yang ^1,2,3,4^

Chia-I Lin ^4,5^

Su-Shin Lee ^6^

Chao-Ling Wang ^4^

Chia-Yen Dai ^1,4^

Hung-Yi Chuang ^1,4,7*^

1. Graduate Institute of Medicine, College of Medicine, Kaohsiung Medical University, Kaohsiung, Taiwan
2. Department of Occupational and Environmental Medicine, Kaohsiung Municipal Siaogang Hospital, Kaohsiung, Taiwan
3. Department of Family Medicine, Kaohsiung Municipal Siaogang Hospital, Kaohsiung, Taiwan
4. Department of Occupational and Environmental Medicine, Kaohsiung Medical University Hospital, Kaohsiung, Taiwan
5. Health Management Center, Kaohsiung Municipal Ta-Tung Hospital, Kaohsiung, Taiwan
6. Center for Stem Cell Research, Kaohsiung Medical University, Kaohsiung, Taiwan
7. Department of Public Health, College of Health Sciences, Kaohsiung Medical University, Kaohsiung, Taiwan

* Corresponding author:

E-mail: [ericch@kmu.edu.tw](mailto:ericch@kmu.edu.tw)

**Supplementary Information**

**Supplementary Method.** Standard procedures of blood lead analysis.

**Supplementary Method.** MT1A and MT2A genotyping procedure.

**Supplementary Table S1**. Demographic analyses of lead levels and biomarkers in participants according to MT1A and MT2A polymorphisms.

**Supplementary Table S2.** Regression model of renal biomarkers predicted by the TWICL and potential confounders without any genotypes adjustment.

**Supplementary Method. Standard procedures of blood lead analysis**

First, the forearms of the participants were washed with soap and fresh water. Then, the antecubital area was cleaned with 70% ethanol cotton balls (no wipe package was used without the possibility of lead-containing foil), and a venous puncture procedure for collecting a 5 ml whole blood sample in a lead-free vacuum tube (Monoject, Sherwood Medical) was arranged. The lead-free vacuum tube was covered with ethylenediamine tetraacetic acid (EDTA) as the anticoagulant. The blood samples obtained for blood lead measurement were analyzed by means of Zeeman effect graphite furnace atomic absorption spectrometry (GF-AAS, Perkin-Elmer 5100 PC with an AS 60 autosampler) after treatment with a 1:4 solution of 0.1% Triton X 100 (Merck, scintillation grade) and 1.25% ammonium dihydrogen phosphate (Merck, puratronic grade) in a class 100 hood, with the air supplied and cleaned by a high-efficiency particulate filter.

**Supplementary Method. MT1A and MT2A genotyping procedure**

Genomic DNA was extracted from peripheral blood using QIAamp DNA Blood Mini Kits (Qiagen, Valencia, CA, USA), and the final preparations were stored at −20°C for use as templates in the polymerase chain reaction. TaqMan Allelic Discrimination assays (Applied Biosystems, Foster City, CA) were used for genotyping, and the samples were treated in a 7300 Real-time PCR System (Life Technologies Corp., Carlsbad, CA). In brief, the genotyping of the MT1A polymorphisms (rs11640851 and rs8052394) and MT2A polymorphisms (rs10636 and rs28366003) were performed by TaqMan SNP genotyping assays (Applied Biosystems, Foster City, CA, USA). The final volume for each reaction was 10 μL, including 5 μL of TaqMan Universal PCR Master Mix, 0.25 μL of primers/TaqMan probe mix, and 10 ng of genomic DNA. The real-time PCR reaction protocol consisted of an initial denaturation step at 95°C for 10 minutes, followed by 40 cycles, each consisting of 92°C for 15 seconds and 60°C for 1 minute. We arranged an Applied Biosystems Step One Real-Time PCR System (Applied Biosystems) to measure the fluorescence level to target genetic sequence detection.

**Supplementary Table S1.** **Demographic analyses of lead levels and biomarkers in participants according to MT1A and MT2A polymorphisms.**

|  | MT1A, rs11640851 |  |  |  |  | MT1A, rs8052394 |  |  |  |
| --- | --- | --- | --- | --- | --- | --- | --- | --- | --- |
|  | AA (n=99) | AC (n=231) | CC (n=155) | p-value # |  | AA (n=249) | AG (n=194) | GG (n=42) | p-value # |
| Age (years) | 42.38 ± 6.92 | 42.73 ± 8.17 | 41.93 ± 8.37 | 0.629 |  | 40.89 ± 7.73 | 43.57 ± 8.10 | 45.98 ± 7.06 | <0.001 * |
| Job duration (years) | 13.74 ± 7.98 | 13.09 ± 7.93 | 12.19 ± 7.47 | 0.280 |  | 11.76 ± 7.22 | 13.91 ± 8.40 | 15.42 ± 7.24 | 0.001 * |
| Body height (cm) | 162.83 ± 7.56 | 161.97 ± 8.18 | 162.39 ± 8.68 | 0.668 |  | 162.70 ± 7.80 | 161.68 ± 8.64 | 162.55 ± 8.52 | 0.420 |
| Body weight (kg) | 61.55 ± 10.61 | 62.43 ± 11.28 | 61.93 ± 11.43 | 0.793 |  | 61.41 ± 11.46 | 62.71 ± 10.80 | 63.21 ± 11.17 | 0.385 |
| Body mass index (kg/m^2^) | 23.16 ± 3.38 | 23.71 ± 3.30 | 23.38 ± 3.29 | 0.351 |  | 23.10 ± 3.38 | 23.93 ± 3.29 | 23.78 ± 2.74 | 0.027 * |
| Systolic blood pressure (mmHg) | 122.94 ± 15.35 | 123.43 ± 17.73 | 17.73 ± 17.05 | 0.958 |  | 122.63 ± 16.23 | 123.77 ± 18.45 | 123.85 ± 14.69 | 0.760 |
| Diastolic blood pressure (mmHg) | 77.03 ± 11.52 | 76.84 ± 10.90 | 76.64 ± 10.73 | 0.961 |  | 76.20 ± 10.79 | 77.46 ± 11.30 | 77.44 ± 10.37 | 0.455 |
| Current blood lead (ug/dL) | 25.64 ± 14.01 | 23.30 ± 13.87 | 18.68 ± 11.20 | <0.001 * |  | 22.08 ± 13.27 | 23.32 ± 13.87 | 18.89 ± 10.65 | 0.139 |
| Index of cumulative lead (ug × yr/dL) | 419.72±364.42 | 344.63±289.06 | 281.63±273.48 | 0.002 * |  | 313.58 ± 287.17 | 373.03 ± 333.74 | 342.01 ± 250.78 | 0.125 |
| Time-weighted ICL (ug/dL) | 28.40 ± 13.14 | 25.73 ± 12.69 | 21.68 ± 11.75 | <0.001 * |  | 24.69 ± 12.57 | 26.02 ± 13.23 | 21.90 ± 10.60 | 0.142 |
| Gender |  |  |  | 0.970 |  |  |  |  | 0.901 |
| Female (%) | 43 (43.4%) | 100 (43.3%) | 69 (44.5%) |  |  | 109 (43.8%) | 86 (44.3%) | 17 (40.5%) |  |
| Male (%) | 56 (56.6%) | 131 (56.7%) | 86 (55.5%) |  |  | 140 (56.2%) | 108 (55.7%) | 25 (59.5%) |  |
| Smoking |  |  |  | 0.955 |  |  |  |  | 0.751 |
| Yes (%) | 38 (38.4%) | 88 (38.1%) | 57 (36.8%) |  |  | 90 (36.1%) | 76 (39.2%) | 17 (40.5%) |  |
| No (%) | 61 (61.6%) | 143 (61.9%) | 98 (63.2%) |  |  | 159 (63.9%) | 118 (60.8%) | 25 (59.5%) |  |
| Drinking |  |  |  | 0.149 |  |  |  |  | 0.724 |
| Yes (%) | 19 (19.2%) | 39 (16.9%) | 17 (11.0%) |  |  | 36 (14.5%) | 31 (16.0%) | 8 (19.0%) |  |
| No (%) | 80 (80.8%) | 192 (83.1%) | 138 (89.0%) |  |  | 213 (85.5%) | 163 (84.0%) | 34 (81.0%) |  |
| Serum uric acid (mg/dL) | 6.40 ± 1.52 | 6.47 ± 1.59 | 6.22 ± 1.46 | 0.283 |  | 6.30 ± 1.46 | 6.51 ± 1.60 | 6.21 ± 1.66 | 0.271 |
| Serum creatinine (mg/dL) | 0.95 ± 0.19 | 0.96 ± 0.24 | 0.94 ± 0.21 | 0.560 |  | 0.96 ± 0.21 | 0.95 ± 0.23 | 0.93 ± 0.22 | 0.532 |
| Urinary creatinine (mg/dL) | 212.11±116.14 | 195.72±98.34 | 167.69±77.86 | 0.001 * |  | 173.18±91.33 | 209.79±102.89 | 199.53±92.48 | <0.001 * |
| Urinary uric acid (mg/g Cr) | 32.71 ± 15.86 | 34.81 ± 16.28 | 42.62 ± 16.19 | <0.001 * |  | 40.13 ± 16.70 | 33.16 ± 15.84 | 34.76 ± 16.18 | <0.001 * |
| Urinary NAG (mg/g Cr) | 3.31 ± 2.18 | 3.14 ± 1.69 | 2.99 ± 1.94 | 0.403 |  | 2.83 ± 1.55 | 3.37 ± 2.06 | 3.76 ± 2.46 | 0.001 * |

**Supplementary Table S1. Continued. Demographic analyses of lead levels and biomarkers in participants according to MT1A and MT2A polymorphisms**

|  | MT2A, rs10636 |  |  |  |  | MT2A, rs28366003 |  |  |  |
| --- | --- | --- | --- | --- | --- | --- | --- | --- | --- |
|  | GG (n = 247) | GC (n = 194) | CC (n = 44) | p-value # |  | AA (n = 381) | AG (n = 101) | GG (n = 3) | p-value # |
| Age (years) | 41.53 ± 8.32 | 43.46 ± 7.63 | 42.66 ± 7.16 | 0.041 * |  | 42.85 ± 7.79 | 40.83 ± 8.50 | 37.64 ± 10.13 | 0.045 * |
| Job duration (years) | 12.43 ± 8.04 | 13.39 ± 7.43 | 13.80 ± 8.03 | 0.327 |  | 13.33 ± 7.81 | 11.54 ± 7.65 | 10.40 ± 9.55 | 0.104 |
| Body height (cm) | 162.28 ± 8.48 | 161.90 ± 8.01 | 163.96 ± 7.49 | 0.324 |  | 162.11 ± 8.19 | 162.80 ± 8.26 | 166.23 ± 9.73 | 0.534 |
| Body weight (kg) | 61.22 ± 11.12 | 62.45 ± 11.55 | 65.38 ± 9.19 | 0.064 |  | 62.16 ± 11.32 | 61.68 ± 10.59 | 66.67 ± 14.98 | 0.721 |
| Body mass index (kg/m^2^) | 23.16 ± 3.27 | 23.73 ± 3.41 | 24.31 ± 2.90 | 0.046 * |  | 23.58 ± 3.44 | 23.16 ± 2.80 | 23.87 ± 2.95 | 0.522 |
| Systolic blood pressure (mmHg) | 121.93 ± 15.71 | 124.26 ± 18.19 | 125.50 ± 18.44 | 0.235 |  | 123.54 ± 17.36 | 121.85 ± 15.85 | 124.33 ± 12.90 | 0.673 |
| Diastolic blood pressure (mmHg) | 76.39 ± 10.57 | 76.74 ± 11.34 | 79.45 ± 11.27 | 0.231 |  | 77.23 ± 10.76 | 75.39 ± 11.73 | 72.33 ± 3.51 | 0.252 |
| Current blood lead (ug/dL) | 18.40 ± 12.10 | 24.73 ± 12.56 | 33.52 ± 14.67 | <0.001 * |  | 23.73 ± 13.29 | 17.18 ± 12.23 | 14.07 ± 14.34 | <0.001 * |
| Index of cumulative lead (ug × yr/dL) | 283.12±279.27 | 364.05±280.55 | 551.34±422.01 | <0.001 * |  | 363.19 ± 312.92 | 255.48 ± 258.10 | 211.63 ± 182.36 | 0.005 * |
| Time-weighted ICL (ug/dL) | 21.33 ± 11.70 | 26.94 ± 11.62 | 36.79 ± 13.88 | <0.001 * |  | 26.31 ± 12.53 | 20.11 ± 12.07 | 19.26 ± 19.07 | <0.001 * |
| Gender |  |  |  | 0.031 * |  |  |  |  | 0.720 |
| Female (%) | 114 (46.2%) | 87 (44.8%) | 11 (25.0%) |  |  | 170 (44.6%) | 41 (40.6%) | 1 (33.3%) |  |
| Male (%) | 133 (53.8%) | 107 (55.2%) | 33 (75.0%) |  |  | 211 (55.4%) | 60 (59.4%) | 2 (66.7%) |  |
| Smoking |  |  |  | 0.039 * |  |  |  |  | 0.626 |
| Yes (%) | 85 (34.4%) | 74 (38.1%) | 24 (54.5%) |  |  | 148 (38.8%) | 34 (33.7%) | 1 (33.3%) |  |
| No (%) | 162 (65.6%) | 120 (61.9%) | 20 (45.5%) |  |  | 233 (61.2%) | 67 (66.3%) | 2 (66.7%) |  |
| Drinking |  |  |  | 0.006 * |  |  |  |  | 0.020 * |
| Yes (%) | 32 (13.0%) | 29 (14.9%) | 14 (31.8%) |  |  | 68 (17.8%) | 7 (6.9%) | 0 (0%) |  |
| No (%) | 215 (87.0%) | 165 (85.1%) | 30 (68.2%) |  |  | 313 (82.2%) | 94 (93.1%) | 3 (100%) |  |
| Serum uric acid (mg/dL) | 6.27 ± 1.48 | 6.41 ± 1.55 | 6.83 ± 1.71 | 0.075 |  | 6.39 ± 1.56 | 6.35 ± 1.48 | 5.73 ± 0.49 | 0.755 |
| Serum creatinine (mg/dL) | 0.95 ± 0.21 | 0.95 ± 0.20 | 1.03 ± 0.35 | 0.082 |  | 0.95 ± 0.23 | 0.96 ± 0.20 | 1.00 ± 0.10 | 0.875 |
| Urinary creatinine (mg/dL) | 180.33 ± 98.23 | 196.10 ± 96.10 | 218.56 ± 95.33 | 0.031 * |  | 198.25 ± 101.51 | 159.68 ± 75.08 | 180.47 ± 83.81 | 0.002 * |
| Urinary uric acid (mg/g Cr) | 40.06 ± 17.43 | 33.90 ± 15.50 | 32.12 ± 13.20 | <0.001 * |  | 34.78 ± 16.48 | 44.66 ± 14.98 | 41.55 ± 13.42 | <0.001 * |
| Urinary NAG (mg/g Cr) | 2.80 ± 1.58 | 3.28 ± 1.86 | 4.28 ± 2.80 | <0.001 * |  | 3.23 ± 1.99 | 2.77 ± 1.34 | 2.17 ± 0.31 | 0.067 |

# p-values calculated from one-way ANOVA tests for continuous variables; x^2^ test for categorical variables.

* p-value <0.05

**Supplementary Table S2.** **Regression model of renal biomarkers predicted by the TWICL and potential confounders without any genotypes adjustment**

|  |  | Serum creatinine (mg/dL) | |  | Serum uric acid (mg/dL) | |  | Urinary uric acid (mg/g Cr) | |  | Urinary NAG (mg/g Cr) | |
| --- | --- | --- | --- | --- | --- | --- | --- | --- | --- | --- | --- | --- |
|  |  | ß (SE) | p-value |  | ß (SE) | p-value |  | ß (SE) | p-value |  | ß (SE) | p-value |
| Time-weighted ICL (ug/dL) |  | -0.0005 (0.0006) | 0.45 |  | 0.0089 (0.0049) | 0.070 |  | -0.20 (0.065) | 0.0018 * |  | 0.021 (0.0070) | 0.0025 * |
| Gender(male) |  | 0.32 (0.021) | <0.0001 * |  | 1.48 (0.16) | <0.0001 * |  | -2.74 (2.042) | 0.18 |  | -0.81 (0.22) | 0.0003 * |
| Age (year) |  | -0.0003 (0.0010) | 0.78 |  | -0.012 (0.0074) | 0.097 |  | -0.075 (0.098) | 0.44 |  | 0.051 (0.011) | <0.0001 * |
| BMI (kg/m2) |  | 0.0012 (0.0024) | 0.60 |  | 0.11 (0.018) | <0.0001 * |  | 0.32 (0.23) | 0.17 |  | 0.043 (0.026) | 0.089 |
| Smoke |  | -0.035 (0.021) | 0.098 |  | -0.054 (0.16) | 0.73 |  | 1.45 (2.098) | 0.49 |  | 0.53 (0.23) | 0.020 * |
| Drink |  | 0.0095 (0.023) | 0.68 |  | 0.041 (0.17) | 0.81 |  | -0.94 (2.29) | 0.68 |  | 0.24 (0.25) | 0.33 |
| Constant |  | 0.78 (0.063) | <0.0001 |  | 3.39 (0.48) | <0.0001 |  | 38.75 (6.29) | <0.0001 |  | -0.36 (0.68) | 0.60 |

* p-value <0.05
